# A scale-free model of acute and ventilator-induced lung injury: a network theory approach inspired by seismology

**DOI:** 10.3389/fnetp.2024.1392701

**Published:** 2024-05-01

**Authors:** Drew C. Gottman, Bradford J. Smith

**Affiliations:** ^1^ University of Colorado School of Medicine, University of Colorado Denver, Aurora, CO, United States; ^2^ Department of Bioengineering, University of Colorado Denver, Anschutz Medical Campus, Aurora, CO, United States; ^3^ Section of Pulmonary and Sleep Medicine, Department of Pediatrics, School of Medicine, University of Colorado Denver, Aurora, CO, United States

**Keywords:** acute lung injury, ventilator-induced lung injury, image segmentation, network theory, scale-free network

## Abstract

**Introduction:**

Acute respiratory distress syndrome (ARDS) presents a significant clinical challenge, with ventilator-induced lung injury (VILI) being a critical complication arising from life-saving mechanical ventilation. Understanding the spatial and temporal dynamics of VILI can inform therapeutic strategies to mitigate lung damage and improve outcomes.

**Methods:**

Histological sections from initially healthy mice and pulmonary lavage-injured mice subjected to a second hit of VILI were segmented with Ilastik to define regions of lung injury. A scale-free network approach was applied to assess the correlation between injury regions, with regions of injury represented as ‘nodes’ in the network and ‘edges’ quantifying the degree of correlation between nodes. A simulated time series analysis was conducted to emulate the temporal sequence of injury events.

**Results:**

Automated segmentation identified different lung regions in good agreement with manual scoring, achieving a sensitivity of 78% and a specificity of 85% across ‘injury’ pixels. Overall accuracy across ‘injury’, ‘air’, and ‘other’ pixels was 81%. The size of injured regions followed a power-law distribution, suggesting a ‘rich-get-richer’ phenomenon in the distribution of lung injury. Network analysis revealed a scale-free distribution of injury correlations, highlighting hubs of injury that could serve as focal points for therapeutic intervention. Simulated time series analysis further supported the concept of secondary injury events following an initial insult, with patterns resembling those observed in seismological studies of aftershocks.

**Conclusion:**

The size distribution of injured regions underscores the spatially heterogeneous nature of acute and ventilator-induced lung injury. The application of network theory demonstrates the emergence of injury ‘hubs’ that are consistent with a ‘rich-get-richer’ dynamic. Simulated time series analysis demonstrates that the progression of injury events in the lung could follow spatiotemporal patterns similar to the progression of aftershocks in seismology, providing new insights into the mechanisms of injury distribution and propagation. Both phenomena suggest a potential for interventions targeting these injury ‘hubs’ to reduce the impact of VILI in ARDS management.

## 1 Introduction

Acute respiratory distress syndrome (ARDS) is a life-threatening condition characterized in part by increased blood-gas barrier permeability, leading to diffuse alveolar damage and severe hypoxemia (Rawal et al., 2018). Approximately 200,000 patients will develop ARDS each year in the United States (Rubenfeld et al., 2005) with an estimated mortality rate of approximately 35%–45% (Bellani et al., 2016). Despite more than 50 years of research, supportive mechanical ventilation to maintain gas exchange remains as the fundamental treatment for ARDS (Banavasi et al., 2021). Mechanical ventilation, while indispensable in managing ARDS, introduces the risk of ventilator-induced lung injury (VILI). This paradoxical phenomenon, where the very treatment designed to support the compromised respiratory system may lead to further lung damage, adds another layer of complexity to ARDS management (Slutsky and Ranieri, 2013).

The pathogenesis of VILI is driven by the mechanical forces of volutrauma (excessive stretch) and atelectrauma (cyclic collapse and reopening) as well as biotrauma due to inflammation. These factors are complicated by the spatial heterogeneity of the lung, which results in an uneven distribution of mechanical forces during ventilation, and is characterized by regional differences in ventilation, perfusion, and mechanics (Herrmann et al., 2022; Mattson et al., 2022; Miserocchi, 2023) that are exacerbated by injury. In some areas, overdistension due to excessive tidal volumes (volutrauma) may predominate, while in other areas, repetitive opening and closing of small airways and alveoli may result in atelectrauma (Bates and Smith, 2018).

Given the complexity and heterogeneity of lung tissue, the pathophysiology of ARDS and VILI have proven difficult to model and predict, resulting in an ongoing challenge to develop effective lung protective ventilation strategies and therapeutic interventions. There has been a recent shift in the field towards understanding these conditions as complex, dynamic systems, an approach that acknowledges the multitude of interconnected factors and feedback loops contributing to the onset and progression of ARDS and VILI (Bates and Smith, 2018). In that context, a hypothesis that has been gaining momentum posits that the progression of VILI is governed by ‘preferential attachment’ or ‘rich-get-richer’ dynamics, where regions of the lung that are initially more damaged are more likely to accrue additional injury over time due to ventilation (Hamlington et al., 2018; Mori et al., 2019). Preferential attachment, and more broadly network theory, has emerged as a powerful tool for studying complex systems across various disciplines, from social networks to biological systems (Czarnecki et al., 2021; Ivanov, 2021; West, 2022), and may offer new insights into the pathogenesis of ARDS and VILI.

In the current study, we further explore the concept of preferential attachment as a driver of the spatiotemporal dynamics of acute and ventilator-induced lung injury. In the context of network theory, preferential attachment posits that new connections within a network preferentially attach to more connected nodes. This leads to a ‘rich-get-richer’ phenomenon, where nodes that are already well-connected become even more central to the network’s structure. A scale-free network is characterized by a few highly connected hubs and many nodes with fewer connections, which is a pattern that emerges from the preferential attachment process. Within the context of our current study, we suggest that this concept of preferential attachment can be recontextualized as a network growth process; that is, areas of injury observed in histological sections are analogous to nodes in a network. These ‘injury nodes’ are interconnected based on factors such as their relative size and proximity. Larger areas of injury are subjected to higher stress and strain and disproportionately affect the propagation of injury throughout the rest of the lung, much like how the wealthiest in an economic system disproportionately affect the rest of the economy or how popular internet sites attract increasing levels of traffic due to their numerous connections. The concept of lung injury as a scale-free network is extended into the temporal domain using a simulated time series, offering a model that could explain changes in the lung over time. By doing so, we can provide insights into how lung injuries may evolve over time, not just as isolated events but as part of an interconnected system. This approach suggests that a small subset of large injury areas exert a disproportionate influence on injury progression. This phenomenon, reminiscent of how wealth accumulates in economic systems or how seismic aftershocks cluster around a main event, offers a compelling analogy for understanding the mechanisms driving the distribution and exacerbation of lung injuries.

## 2 Methods

### 2.1 Animal procedures

The current study is a secondary analysis of images generated in previously reported experiments (Bilodeaux et al., 2023) and the key experimental details are summarized below. The study was conducted on female C57/BL6 mice, aged seven to 10 weeks, and was approved by the University of Colorado Denver Institutional Animal Care and Use Committee (IACUC) under protocol #00230. A lavage group (LAV) of mice were subjected to injury by administering an intratracheal instillation and suction of 0.15 mL saline just before ventilation, while the control group (CTRL) was not subjected to any injury.

The mice were anaesthetized through an intraperitoneal injection of 100 mg/kg ketamine, 8 mg/kg xylazine, and 2.5 mg/kg acepromazine, a tracheostomy was performed, and respiratory muscle activity was halted by giving the mice a 0.8 mg/kg dosage of pancuronium bromide at the commencement of mechanical ventilation.

The LAV group underwent 25 min of injurious ventilation at an inspiratory pressure of 37.5 cmH2O and a positive end expiratory pressure (PEEP) = 0. The control group received 6 minutes of stabilizing low tidal volume ventilation at PEEP = 3 cmH_2_O. All groups then received a series of lung function measurements with PEEPs ranging from 0 to 15 cmH_2_O as detailed elsewhere (Bilodeaux et al., 2023). Total ventilation time was ≈35 min for CTRL and ≈60 min for LAV.

After ventilation, a bilateral thoracotomy was performed and the pulmonary circulation was flushed with heparinized saline. After performing three recruitment maneuvers, the airway pressure was sustained at 30 cmH_2_O for 3 seconds before being reduced to two cmH2O and ligating the trachea. The lungs were then perfused through the vasculature with 5 mL of fixative and then immersion fixed for 24+ hours. After post-fixing in osmium tetroxide and uranyl acetate and embedding in glycol methacrylate the tissues were sectioned at 1.5 μm and stained with toluidine blue.

### 2.2 Image segmentation

Whole slide images were captured using a ×20 objective and imported into Ilastik version 1.4 (Berg et al., 2019). Ilastik’s random forest classifier was iteratively trained on manual pixel-level annotations, marking regions as ‘air’, ‘injured’, or ‘other’. The training process was conducted through Ilastik’s graphical user interface, which allows for real-time feedback by displaying the classifier’s predictions overlaid on the original images. The annotator (DG) adjusted these predictions by directly correcting misclassified pixels, refining the classifier’s accuracy through successive iterations until the segmentation appeared suitable.

We define ‘air’ as regions devoid of tissue but within the lung, including large vessels that were cleared of blood during the perfusion fixation. ‘Injured’ referred to regions of the parenchyma with atelectasis, as indicated by multiple layers of septal capillaries ‘piled up’ (Wallbank et al., 2023), or airspace edema. ‘Other’ was defined to include all other tissue including patent septa, airway walls, and interstitial space. Training data comprised 12 whole-slide images, equally distributed between the CTRL and LAV groups. 47 distinct features were used for pixel classification including color, edge, and intensity variations across scales from 2 to 100 pixels, and edge and texture details at a finer scale of 0.7 pixels. The full set of features is provided in Supplementary Table S1.

Pixel classifications were post-processed in MATLAB version 2022a, the segmentation performance was manually scored, and the remainder of the data analysis was conducted.

Air and extraneous image artifacts found outside of the lung were segmented with k-means clustering and excluded from the analysis. Images containing multiple lobes were separated by manually creating image masks to allow single-lobe analysis. A mask was created by first identifying pixels classified as ‘other’ and creating separate masks for contiguous regions more than 100 pixels from each other. Masks smaller than 100000 pixels correlated with noisy artifact and were removed. Lobes within 100 pixels of each other were then manually separated from each other to create distinct masks for each lobe. Extraneous artifacts greater than 100000 pixels in size that did not constitute a lobe also had their masks removed. Finally, holes in each mask corresponding to large airways were closed.

Injured regions (injury nodes) were defined from the 8-connected islands of ‘injured’ class pixels. Injured regions of less than 100 contiguous pixels (52.77 μm^2^) were deemed to be noise and were removed from the image. Injured pixels were then processed with morphological dilation and then erosion five times, each using a five-pixel wide disk-shaped structuring element, to merge injured regions in close proximity to each other. In our network analysis, distinct ‘injured’ nodes were delineated based on these processed segments, with any interruption by non-‘injured’ pixels serving to define the boundaries of individual nodes.

To determine the accuracy of the segmentation, 20 pixels from each post-processed segmentation class (‘injured’, ‘air’, and ‘other’) were chosen randomly for each whole-slide image and manually scored by a reviewer (DG). This comparison aimed to establish the ground truth for injury pixels within a subset of our images. Sensitivity and specificity were calculated for each segmentation class to quantify the accuracy of our automated method in correctly identifying each class of pixels as compared to a human reviewer.

### 2.3 Scale-free modeling

Following image segmentation, scale-free modeling of regions of injury, which are defined as airspace edema and atelectasis, was performed on the segmented whole-slide images. This approach is adapted from a scale-free model previously used in seismology. This approach, which correlates main shocks and aftershocks in both spatial and temporal dimensions, relies on a power-law distribution to describe the number of correlations that an injured region may receive from other injured regions (Baiesi and Paczuski, 2004). The rationale behind choosing a power-law distribution over exponential, or other types, arises from the observation that in competitive environments, like the stressed lung, resources, or, in this case, the lung’s resilience to injury, are not uniformly distributed. Thus, certain regions, akin to ‘hubs’ in a network, disproportionately accumulate more damage. This phenomenon, known as preferential attachment or the ‘rich-get-richer’ dynamic mirrors phenomena observed in complex networks across various fields, from ecology to seismology to economics, and has been previously shown to describe the injured lung (Hamlington et al., 2018; Mori et al., 2019; Gaver et al., 2020).

We first start with the proposition that sizes of injury follow a power-law distribution, which has been empirically demonstrated in previous literature and is recapitulated in the current study (Hamlington et al., 2018; Mattson et al., 2022). Accordingly, the distribution of the number of areas of injury (*P*) of size m in a given region is

$$P\left(m\right)\;∼\;m^{-\alpha },$$ (1)

with α fit to the segmentation data using maximum likelihood estimation. Using the distribution described in Eq. 1, the average number of areas of injury $\bar{n}$ within an interval ∆m of m , occurring in a radius r over a time interval τ , is

$$\bar{n}=C∙\tau ∙r^{d_{f}}∙∆m∙m^{-\alpha },$$ (2)

with $d_{f}$ representing the fractal dimension fitted according to a box-counting algorithm and C a scaling constant. The box-counting algorithm is a technique for estimating the fractal dimension of injured regions by overlaying the lobe mask with a grid of boxes. The size of the boxes is systematically reduced by powers of two, and the number of boxes needed to cover all of the injured regions in a given lobe is counted. By plotting the logarithm of the box count against the logarithm of the inverse of the box size, a linear relationship emerges. The slope of this line represents the fractal dimension, which captures how densely injured regions fill a given two-dimensional region in the lobe. Since it is not possible to obtain time-dependent samples in the same rodent, Eq. 2 is modified remove the time dependence:

$$\bar{n^{*}}=C∙r^{d_{f}}∙∆m∙m^{-\alpha }.$$ (3)

Next, we consider an area of injury denoted by j and we consider how to correlate it with any arbitrary area of injury denoted by i . Given Eq. 3, the expected number of injured areas that are expected to occur within ∆m of $m_{i}$ and in the area bounded by i and j is,

$$n_{ij}^{*}=C∙l^{d_{f}}∙∆m∙m^{-\alpha },$$ (4)

where $l=l_{ij}$ is equal to the Euclidean distance between the centers of regions i and j . The injury i that most strongly correlates with injury j is injury $i^{\prime }$ such that $n_{ij\;}^{*}$ is minimized. A smaller $n_{ij\;}^{*}$ means that the average number of injuries between i and j is lower, indicating that these two injured regions are likely associated and did not occur by random chance alone. Note that Eq. 4 allows us to correlate injured areas together without any assumptions of the generative mechanisms of injury propagation.

In terms of network theory, Eq. 4 provides a network of nodes and links, with nodes representing areas of injury and links, directed from injured region i to injured region j, representing correlations between nodes. We use the simplest implementation of the network, which is for each node j we attach a single link to node $i^{\prime }$ that minimizes $n_{ij\;}^{*}$ and simplify the notation to $n_{j^{*}\;}^{*}$ . Other links for node j are discarded. This provides us with a hierarchically organized cluster of injured areas, with smaller $n_{j^{*}\;}$ denoting a stronger correlation. The in-degree of node i is the number of regions j that correlate to it. We demonstrate that the in-degree distribution (the distribution of the degrees throughout the network) follows a power law. A network with a power law in-degree distribution is also referred to as a scale-free network.

The model formulation described above allows us to correlate areas of injury across space. However, it is not possible to correlate the injured areas through time because our experimental measurements of injured regions require a terminal procedure. To overcome this experimental constraint Eq. 4 is amended to correlate areas of injury across space and time:

$$n_{ij}=C∙l^{d_{f}}∙t_{ij}∙∆m∙m^{-\alpha },$$ (5)

with $t_{ij}$ representing the simulated time interval between i and j and $n_{ij}=n_{ij}^{*}*\;t_{ij}$ . Thus, it is necessary to prove the in-degree distribution of the network formed by minimizing $n_{ij}$ is also power-law distributed given that $n_{ij}^{*}$ is power-law distributed. A proof that the network derived using Eq. 5 is scale-free and provided in the Supplementary Material.

### 2.4 Centrality

In order to designate areas of injury that were considered important in the context of the overall network the concept of centrality from graph theory was used. Indicators of centrality assign numbers or rankings to nodes in a graph corresponding to their importance in the graph. Numerous centrality measures exist. In the current study we use PageRank centrality, a version of eigenvector centrality originally developed for Google’s ranking of webpages in their search results (Brin and Page, 1998). Eigenvector centrality ranks nodes based on the number of connections that they have and the centrality scores of the nodes that they are connected to. PageRank modifies this by introducing a regularization term that ensures that each node has a baseline centrality score and that high-scoring nodes have an upper bound that prevents runaway centrality scores.

### 2.5 Statistical analysis

Data was curated and analyzed in MATLAB version 2022a. Differences in median fraction of injured area between CTRL and LAV samples were assessed by bootstrapping 10,000 samples and counting the proportion of bootstrapped difference of medians that exceeded the actual difference in median. This same method was used to assess for difference in mean and to create 95% confidence intervals for other analyses.

Assessment of power-law fit in our analysis was done through combined maximum-likelihood fitting and Kolmogorov-Smirnov (KS) goodness-of-fit tests with p≥0.1 signifying that a power-law distribution is a plausible fit for the data (Clauset et al., 2009). The null hypothesis in this case is that a power-law function could plausibly explain the distribution of the data; thus, higher *p*-values indicate a greater likelihood that the distribution follows a power law. Injury size, degree distribution of the network with and without synthetic time points, and a theoretical analysis of secondary injury event rates were all assessed using KS goodness-of-fit tests.

For each lobe in our analysis, a time-point was randomly assigned to each injured region with inter-temporal intervals drawn from an exponential distribution with a mean inter-injury rate of 2 seconds. The exponential distribution is chosen for illustrative purposes because it is the probability distribution for time between events in a Poisson point process. This was repeated 100 times to create a distribution of possible values for each analysis. 10,000 bootstrapped samples were used to assess for statistically significant differences of means or medians and to create 95% confidence intervals for analysis involving repeated temporal simulations. To capture secondary injury event rates across varying time frames, we employed exponentially increasing time bins, allowing for a more uniform sampling of event rates over time. When relevant, multiple comparisons were corrected for using the Benjamini–Hochberg procedure.

## 3 Results

### 3.1 Morphometric analysis

**FIGURE 1:**
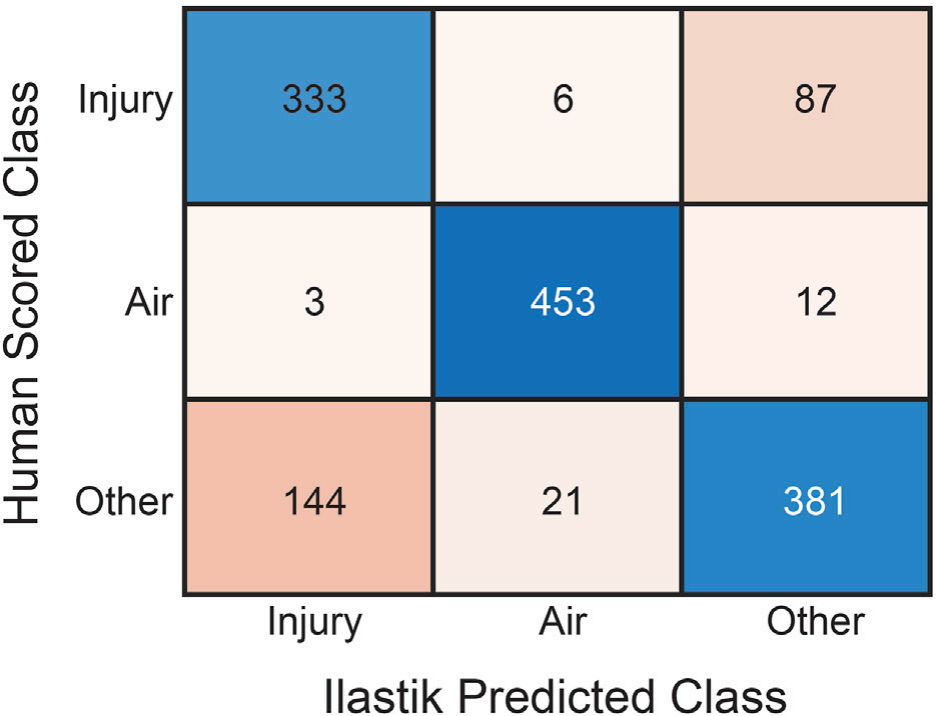
Confusion matrix of human classification *versus* automated pixel classification using Ilastik for the three pixel classes: ‘Injury’, ‘Air’, and ‘Other’. Rows are human scores while columns are Ilastik scores. For each tissue section, twenty pixels from each class were manually scored.

Twenty pixels from each of the three Ilastik-identified classes (‘injury’, ‘air’, and ‘other’) for each image were manually scored using the post-processed segmentations as shown in the confusion matrix (Figure 1). The automated classification of ‘injury’ pixels demonstrated a sensitivity of 0.78, indicating that 78% of true ‘injury’ pixels were correctly identified when compared to human scoring. Specificity was 0.85, indicating that 85% of non-injury pixels were correctly excluded. For the ‘air’ class, both sensitivity and specificity were 0.97. For the ‘other’ class, which encompasses a range of tissue types, the sensitivity was 0.69 and the specificity was 0.89. The overall accuracy across all classes was 0.81.

**FIGURE 2:**
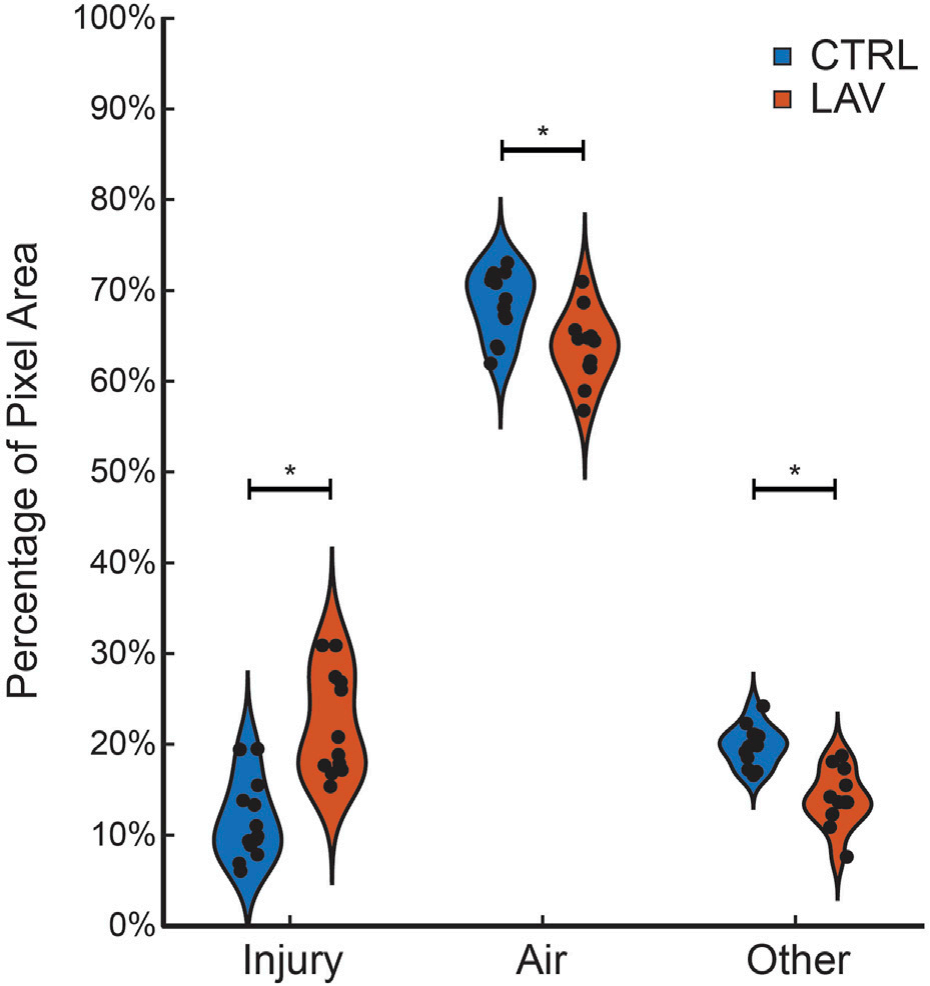
Fraction of ‘Injury’, ‘Air’, and ‘Other’ pixels in the tissue sections for the CTRL (blue) and LAV (orange) groups. Points show individual sections. Statistically significant difference of means between CTRL and LAV are denoted with an asterisk (*p* < 0.05).

**FIGURE 3:**
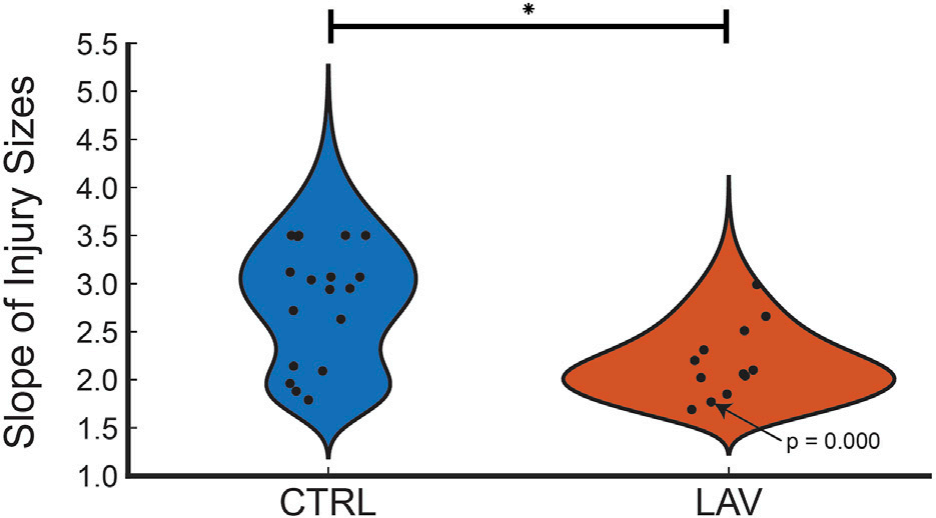
Distribution of power law slopes of the frequency of injury sizes for CTRL and LAV files. The one tissue section that did not fit a power law distribution is annotated with the *p*-value. The significant difference between CTRL and LAV is denoted with an asterisk (*p* < 0.05) indicates a greater frequency of large regions of injury in LAV.

The percentage of the lung lobe area occupied by each pixel class in the CTRL and LAV groups is shown in Figure 2. Notably, the median injury extent of 19.82% in the LAV group is significantly elevated compared to the 9.88% in the CTRL group. The LAV group also demonstrated a significantly reduced fractions of air and ‘other’ tissue (which includes aerated septa) compared to CTL. Based on our prior work (Hamlington et al., 2018; Mattson et al., 2022) we hypothesized that the distribution of injured region sizes would follow a power-law distribution and that the power law exponents would differ between the two injury groups. All tissue sections except for one sample in the LAV group demonstrated a power-law distribution of injury sizes (Figure 3). The power-law slope of the histogram of injury sizes was calculated and shows that the median slope for LAV was significantly less than for CTRL, indicating a higher prevalence of larger injury areas in LAV than CTRL.

### 3.2 Scale-free network

**FIGURE 4:**
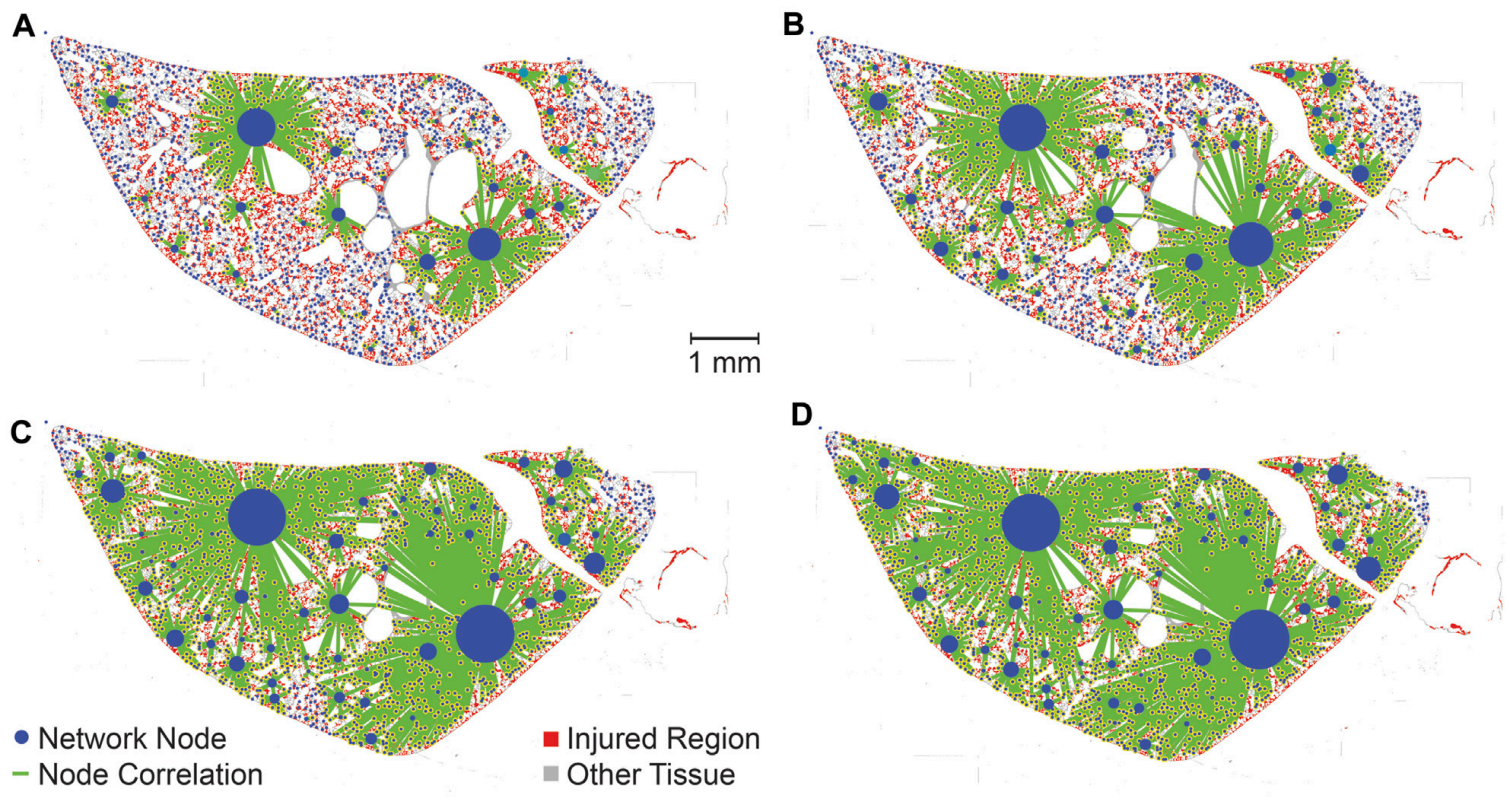
The strength of correlations between different injured areas are elucidated by varying the threshold *n*_*c*_ according to percentiles of $n_{j^{*}\;}^{*}$ . **(A)** has a threshold at the 25th percentile, **(B)** at the 50th percentile, **(C)** at the 75th percentile, and **(D)** at the 90th percentile. Injured pixels are red, while other tissue is grey. Nodes of the network, corresponding to discrete, injured regions, are denoted with a blue circle and green lines represent correlations between nodes in the network. The number of correlations that a given injured region receives is directly proportional to the size of the blue circle representing that injured region.

**FIGURE 5:**
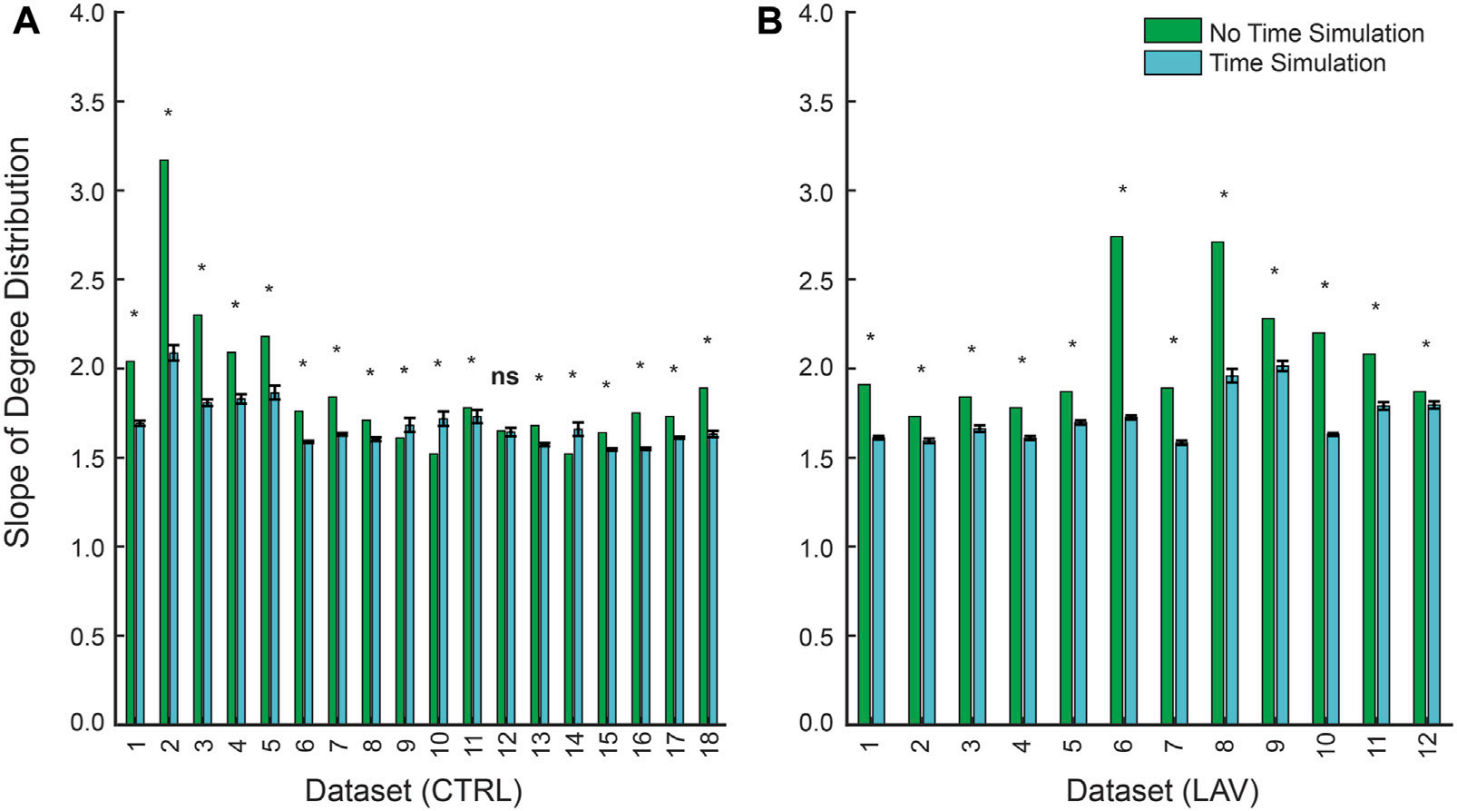
Slopes of power-law fits for the in-degree distribution between non-time simulated and time simulated scale-free networks for **(A)** CTRL and **(B)** LAV. Error bars represent 95% CI for slopes of the in-degree distribution for the time simulated dataset. Significant differences ( p<0.05 ) between the slope for the in-degree distribution between no-time simulation and time-simulation are denoted with an asterisk while differences that are not significantly different ( p≥0.05 ) are denoted with ‘ns’. All samples included in this analysis were found to have in-degree distributions that could be plausibly fitted with a power-law distribution.

The link weights ( $n_{j^{*}\;}^{*}$ ) serve as a measure of the correlation strength between nodes *i* and *j* in the network model. Smaller $n_{j^{*}\;}^{*}$ indicate stronger correlations. The in-degree distribution of the network, which is indicative of the number of correlation, is scale-free: the in-degree distribution of the network ( x ) fits a power-law distribution $P\left(x\right)=x^{-\alpha },$ where *α* is the slope (Figure 5). To elucidate the nature of these correlations a threshold value ( $n_{c}$ ) is used to exclude weaker node correlations with $n_{j^{*}\;}^{*}>n_{c}$ . This approach reveals a spectrum of network structures, ranging from a larger, interconnected network with many weaker correlations, to a decomposition of the network into isolated clusters with stronger correlations (Figure 4). Choosing $n_{c}$ at the 25th percentile of link weights demonstrates a network composed of many isolated injured regions with only strong correlations linking injured regions together. At the 50th percentile, more correlations emerge, while $n_{c}$ at the 75th percentile shows little difference from a threshold at the 90th percentile. Hubs are observed at every $n_{c}$ percentile, where a ‘hub’, has a disproportionate number of correlations compared to other nodes in the network. As the threshold is increased, hubs tend to disproportionately gain more correlations compared to other nodes in the network, suggesting a ‘rich-get-richer’ phenomenon.

**FIGURE 6:**
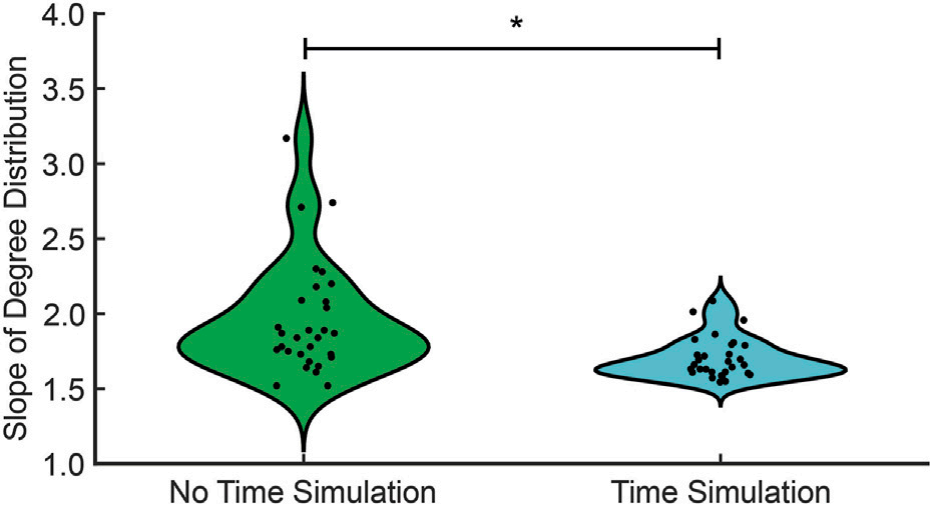
The in-degree distribution for no time simulation (green) and time simulation (cyan). The significant difference in the median between no time simulation and time simulation is denoted with an asterisk ( p<0.05 ).

We next consider the distribution of link weights using repeated temporal simulation. As in the no-time simulation analysis, the temporal simulations follow a scale-free network as indicated by in-degree distributions ( k ) that fit a power-law distribution $P\left(k\right)=k^{-\gamma }$ , where γ is the slope fitted from maximum likelihood estimation (Figure 5). For most samples, γ was significantly greater in the no-time simulations than in the temporal simulations leading to a significantly higher median γ (Figure 6).

**FIGURE 7:**
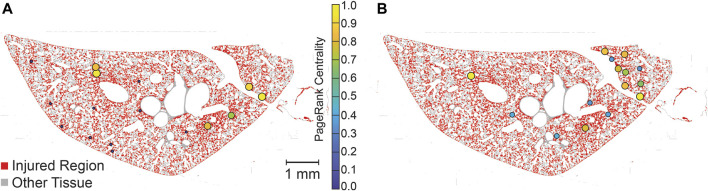
Visualization of PageRank centrality of injured regions with no-time simulation **(A)** and time simulation **(B)**. PageRank centralities are shown if they are in the 99th percentile for magnitude. Centralities are normalized for each lobe (two lobes, analyzed separately, are shown in each image), allowing for comparison within lobes but not between lobes. Injured pixels are red while healthy tissue pixels are grey.

The PageRank centrality was calculated for each lobe, both with and without temporal simulations, to identify the most influential nodes, or injured regions, within the network. A representative lobe is depicted in Figure 7 showing the no time simulation (A) and time simulation (B) analyses. While this figure does not provide a quantitative comparison between the ‘no time simulation’ and ‘time simulation’ analyses, it allows visual comparison of influential nodes between the two analyses.

**FIGURE 8:**
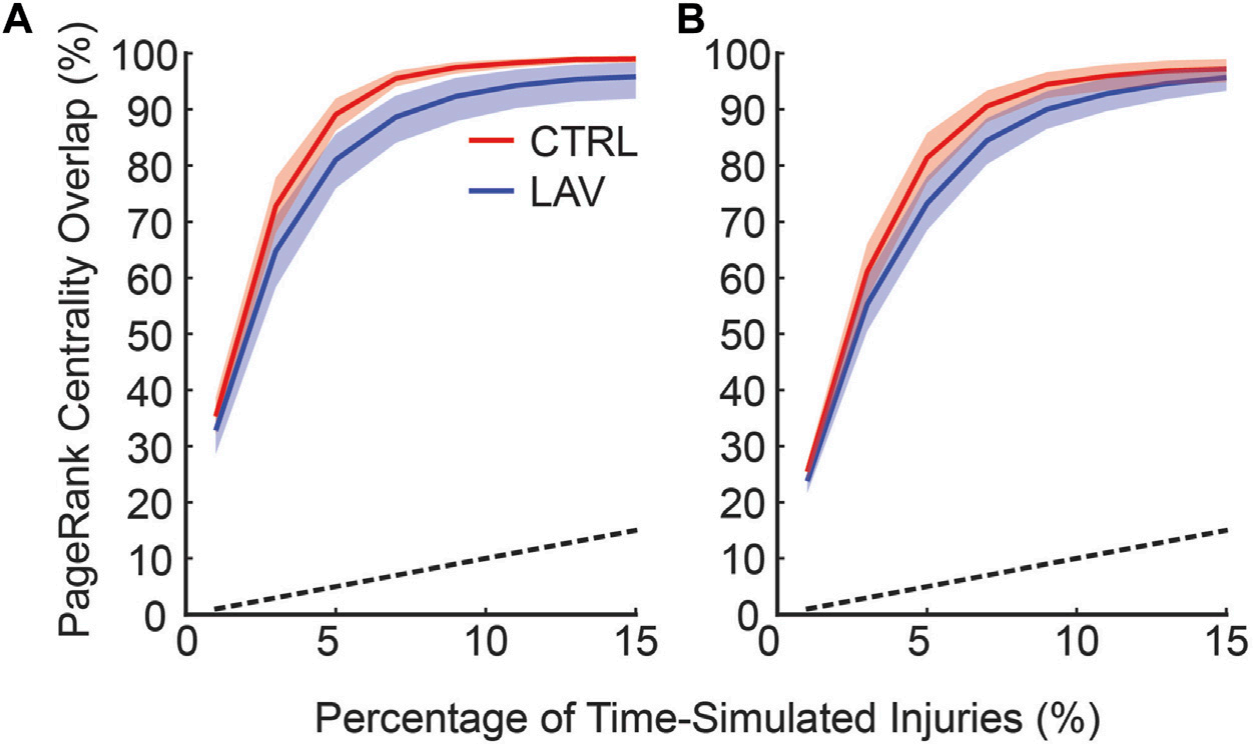
The frequency at which injuries with top centrality scores in the no time simulations are found in the set of injuries with top centrality scores and temporal simulation. The horizontal axis shows the fraction of the injured areas with the highest centrality scores in the time simulation analysis; the vertical axis denotes the fraction of injured areas in the no time simulation analysis that are found in the subset of injured areas in the time simulation analysis (i.e., overlap). **(A)** shows the top 0.5% of injured areas based on centrality scores in no time simulation analysis while **(B)** shows the top 1% of injured areas from the no time simulation analysis. Error bars are 95% CI capturing inter-lobar variability. The black dashed line indicates the percentage overlap that would occur by chance alone.

To quantitatively compare of the consistency between the time and no-time simulation centrality scores two subsets of no-time simulation injured areas were generated using the top 0.5% (Figure 8A) and top 1% (Figure 8B) of scores. The overlap between the no-time simulation and increasing percentages of the top time simulated centrality scores is shown in Figure 8. This analysis revealed that injured areas with the highest centrality scores in the no time simulation analysis frequently corresponded to those with high scores in the time simulation analysis. The degree of overlap increased as the size of the subset in the time simulation analysis expanded. For example, approximately 90% of the top 0.5% injured areas in the no time simulation analysis were found within the top 8% of the time simulation analysis (Figure 8A). Similarly, about 90% of the top 1% of injured areas in the no time simulation analysis were identified within the top 10% of the time-dependent analysis (Figure 8B). The overlap for both CTRL and LAV was significantly greater than chance alone, denoted by the dashed black line (Figure 8). While the LAV group visually appears to show better agreement, the mean difference in overlap between the two groups was not statistically significant, except for a few instances as indicated by confidence bands in Figure 8A.

**FIGURE 9:**
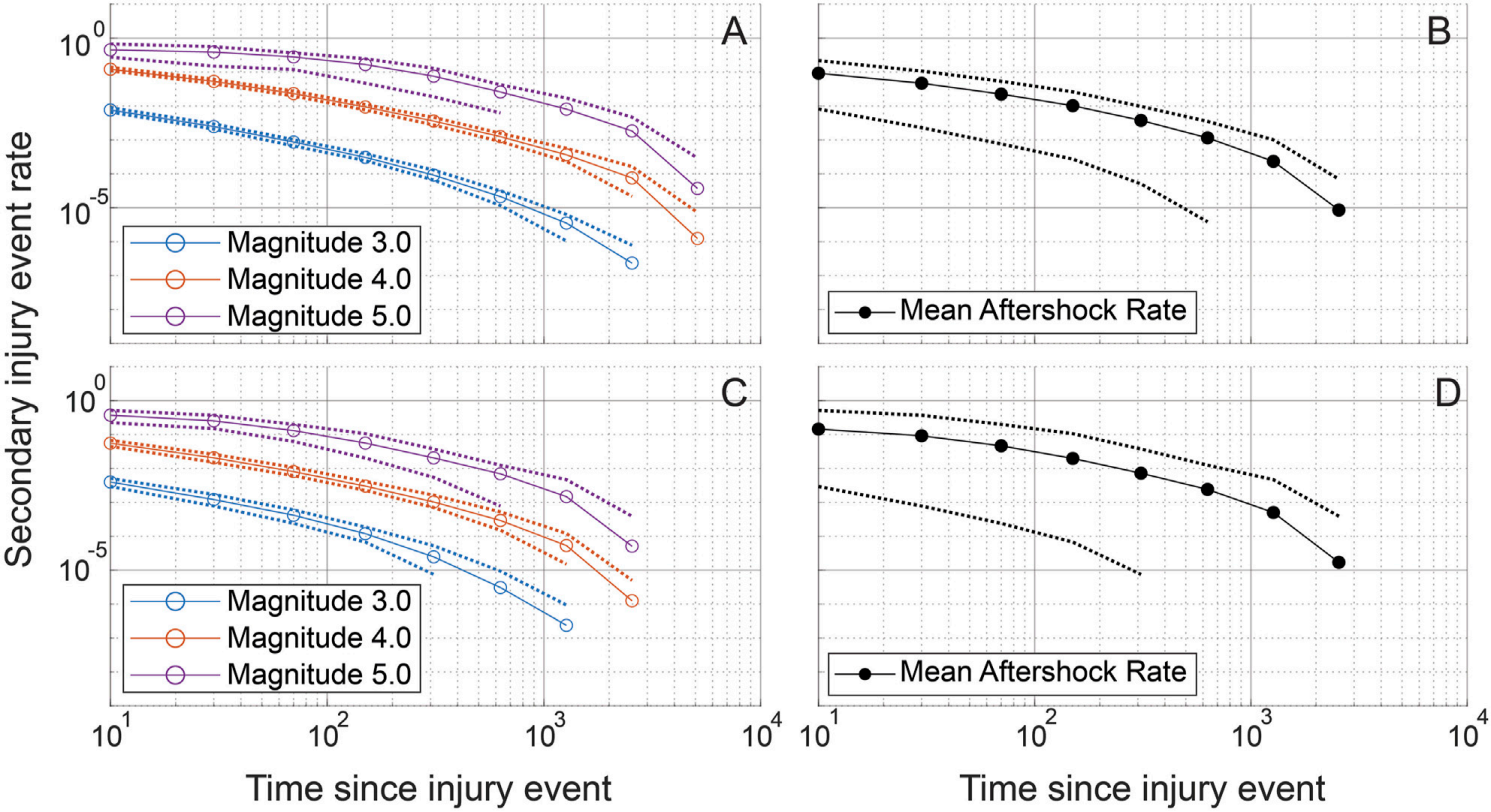
Simulated secondary injury event rates after a primary injury event are shown for randomly chosen CTRL **(A, B)** and LAV **(C, D)** sections. Simulated secondary injury event rates are shown separated by magnitude **(A, C)** and not separated by magnitude **(B, D)**. 95% confidence bands are plotted for each magnitude and for the aggregated magnitudes. Injury magnitudes (sizes) are shown in a log_10_ scale, analogous to the Richter scale, with a reference injury size of 1. Secondary injury event rates approximate power-law behavior with clear separation between secondary injury event rates at different magnitudes. Exponential binning is used to obtain approximately equal sample sizes in each time bin.

Using the time simulation analysis, we next consider the rate of secondary injury occurrence related to primary injury events. This analysis was inspired by Omori’s law in seismology, drawing a parallel between secondary lung injuries and seismic aftershocks. Here, a secondary injury event is defined as one that is correlated with a preceding primary injury event in the temporal simulations. Because the order of injury is randomly generated each temporal simulation was repeated 100 times and ensemble averages are reported. The simulated secondary injury event rate shown for representative CTRL and LAV lobes is provided in Figure 9 and shows that the rate of secondary injury events tends to follow an approximate power-law behavior relative to the time elapsed after primary injury events. Notably, this rate is also influenced by the magnitude of the primary injury event, with larger primary injury events demonstrating statistically significant higher rates of secondary injury events. Although LAV lobes display a significantly higher percentage of injury pixels compared to CTRL lobes, both groups exhibit a similar power-law distribution in the temporal pattern of their secondary injury event rates.

**FIGURE 10:**
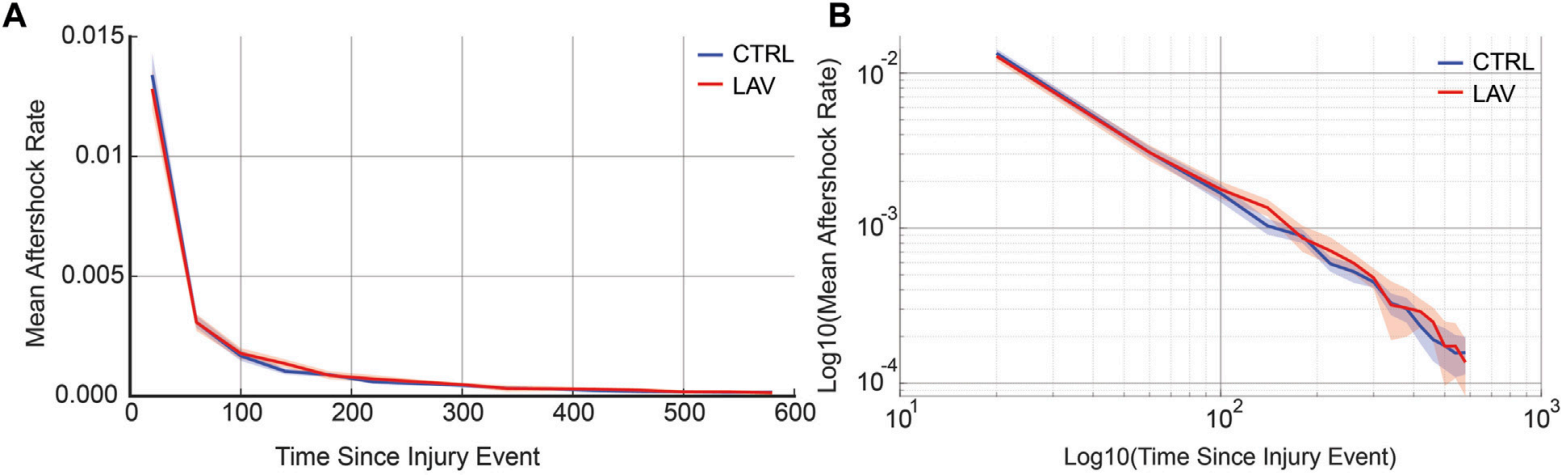
Aggregate secondary injury event rates for the simulated temporal analysis are shown for CTRL and LAV on both a **(A)** linear and **(B)** log-log scales. 95% confidence bands are also shown. Secondary injury event rates demonstrate power-law behavior with no significant difference between CTRL and LAV throughout the entire simulated duration of secondary injury event rates.

An aggregate analysis of aftershock rates, encompassing both CTRL and LAV groups, reveals that the distribution of secondary injury event rates adheres to a power-law distribution. Interestingly, there was no significant difference observed between the CTRL and LAV groups in terms of secondary injury event rates, as shown in Figure 10.

## 4 Discussion

While mechanical ventilation is a necessary, life-saving treatment in patients with ARDS, it poses a risk of further lung damage. This study aims to better understand the spatiotemporal dynamics of a murine two-hit model of acute and ventilator induced lung injury. Using algorithmically segmented images, we apply a network theory approach inspired by prior seismology work to describe how disparate regions of injury are correlated to one another. We found that the distribution of injury sizes in segmented, whole-lobe histological sections follows a power-law distribution, recapitulating findings from our prior work (Hamlington et al., 2018; Mattson et al., 2022). The network theory-based analysis revealed that correlations between separate regions of injury follow a scale-free network, suggesting a ‘rich-get-richer’ phenomenon. This approach was extended using repeated simulations of the temporal dynamics for each whole-slide image. The network remained scale-free with the simulated time-points, with a subsequent exploratory analysis finding that secondary injury event rates follow a power-law with clear delineation between magnitudes, analogous to Omori’s law from seismology. The analogy to seismic aftershocks not only illustrates the cascading nature of lung injury but also underscores the competitive environment within the lung, where larger injury sites are more prone to expansion. Understanding that larger injury sites are more likely to expand provides insight into the spatiotemporal progression of VILI and suggests that ‘hubs’ of injury could become important targets for therapeutic interventions.

The cornerstone of the analysis is pixel classification, which was performed in Ilastik. Comparison to manual classification (Figure 1) shows that the sensitivity for ‘injury’ pixels was 0.78, indicating that automated segmentation and the morphological post-processing steps accurately describes ‘injury’ pixels, while the specificity of 0.85 indicates that most non-injury pixels are correctly excluded. In a prior study a different observer (BJS) performed manual morphometry (stereology) on the same samples (Bilodeaux et al., 2023) and found that the percentage of collapsed septal tissue in the entire lung was 14.55% for LAV and 2.28% for CTRL. In the current study the automated approach yielded 19.82% in the LAV group and 9.88% in the CTRL group. Some of this bias towards the injury classification may be attributed to the different observers in the two studies. This is particularly relevant when differentiating a region of atelectasis (i.e., injury), which is characterized by two or more layers of septal capillaries ‘piled up’ (Rizzo et al., 2021), from an image region where the section plane passes in parallel through the septa and the intra-septal capillary network is revealed (a ‘tangential cut’). While our automated segmentation method demonstrates high sensitivity and specificity compared to a trained expert, it is important to consider the implications of misclassification. Misclassification could impact the interpretation of injury distribution and progression patterns, potentially affecting the identification of injury ‘hubs’ and the application of scale-free analysis. However, given the likelihood that regions misclassified as injury (instead of healthy septa) are ‘tangential cuts’ of the alveolar septa, and thus have a small size relative to other injured regions and a relatively homogenous distribution throughout the lungs, we expect that overestimation of those injured regions does not substantially affect the network analysis. Furthermore, this issue should affect both the LAV and CTRL groups equally, thus minimizing its impact on the comparative analysis of our results.

Prior studies suggest that the pathophysiology of ARDS may be driven, in part, by a ‘rich-get-richer’ phenomenon, wherein larger injured regions expand more rapidly during ventilation. This conceptual framework yields a distribution of injury sizes that follows a power-law distribution (Hamlington et al., 2018; Mori et al., 2019; Gaver et al., 2020; Mattson et al., 2022) and has been demonstrated to exist at scales ranging from perforations in the blood-gas barrier (Hamlington et al., 2018) through cellular injury patterns at the lobar scale (Mori et al., 2019; Gaver et al., 2020; Mattson et al., 2022). On a global scale, computed tomography (CT) scans of chronic obstructive pulmonary disease (COPD) patients have found that the volumes of low attenuation regions, correlating to the presence of lung disease, also follow a power-law distribution (Mondonedo et al., 2019). In the current study we also found that the sizes of injured regions, primarily comprised of microatelectases, followed a power-law distribution (Figure 3). The inter-animal heterogeneity within groups (Figure 3), may also suggest that this scale-free behavior extends through the animal scale.

Our network analysis, without time dependence, lends additional support to this ‘rich-get-richer’ idea. Note that we are performing this analysis using histological sections so repeated measurements in the same animal are not possible. Qualitatively, we observed a distinct hierarchy within the lung injury network, where certain regions, acting as ‘hubs’, were disproportionately more correlated with other injured areas (Figure 4). Upon introduction of a threshold to eliminate weaker correlations, these ‘hubs’ tend to retain more of their correlations than less-connected injured regions, reinforcing the ‘rich-get-richer’ dynamic found in previous literature (Hamlington et al., 2018; Mori et al., 2019; Gaver et al., 2020). Quantitatively, we find that the network’s in-degree distribution adheres to a power-law (Figure 5). In other words, most injured areas are only minimally interconnected, while a few key regions exhibit extensive correlations with other injured areas, reinforcing the ‘rich-get-richer’ pattern. The concept of injury hubs underscores the non-uniform nature of lung injury progression, where certain regions become critical nodes of damage amplification. Biologically, these hubs could be areas of heightened vulnerability within the lung, possibly due to pre-existing microstructural heterogeneities, differential exposure to mechanical stress during ventilation, or variations in local immune responses. Once established, these hubs are likely to propagate injury by generating mechanical stress concentrations. Such a pattern aligns with the scale-free behavior observed at smaller scales and highlights potential targets for therapeutic interventions. Protecting these hubs, or mitigating their expansion, could disrupt the ‘rich-get-richer’ dynamic, potentially halting the progression of lung injury at a critical juncture. This approach could involve strategies such as personalized ventilator management to eliminate stress concentrations or localized delivery of anti-inflammatory agents, pulmonary surfactant, antioxidants, or therapies designed to enhance tissue repair mechanisms specifically within or around these hubs. The development of such targeted interventions necessitates a multidisciplinary approach, combining insights from bioengineering, pharmacology, and clinical medicine to translate our understanding of injury hubs into practical treatments for ARDS and VILI. Perhaps most important is the concept of *preventing* formation of the injury-driving hubs before the form through preemptive lung protective ventilation (Brower et al., 2000; Jabbari et al., 2013; Nieman et al., 2023).

The temporal dynamics of injury are a key concern in the management and treatment of lung injury. However, high-resolution serial imaging of lung parenchyma remains challenging due to methodological constraints. To bridge this gap, we employed a simulated time series analysis. Here, our motivation was to explore the concept of secondary injury events which could be viewed as analogous to seismic aftershocks, where initial injury events could trigger subsequent damage in a cascading temporal sequence. Before analyzing the secondary injury events, we compared PageRank centrality scores for injured regions without the time series and with an ensemble of 100 time series simulations as shown for a pair of sections in Figure 7, which qualitatively demonstrates good overlap between influential regions of injury. This overlap between the two analyses is quantified in Figure 8 showing that the simulated time series, while a constructed model, aligns closely with the non-simulated time series. Using the simulated time series we investigated the concept of secondary injury events, inspired by the analogous phenomena observed in seismology (Yukutake and Iio, 2017). Similarly to seismic aftershocks, we find that secondary injury events in the simulated time series follow a pattern similar to Omori’s law, with clear delineation of secondary injury event rates by magnitude and secondary injury event rates that follow power-law behavior over time (Figure 9). Despite inter-group differences in injury region size, we observe no difference in the secondary injury event rate between CTRL and LAV (Figure 10). This suggests that despite differences in initial injury, ventilation, and injury severity the dynamics of injury propagation remain similar between groups.

This concept of injury aftershocks extends Mead’s concept of parenchymal stress concentrations (Mead et al., 1970) beyond the boundaries of the original injury site and further into the parenchyma. Parenchymal tethering, or alveolar interdependence, occurs because alveoli share septal walls. As such, the collapse or stiffening of one alveolus will increase stress and strain in neighboring regions. This theory has been bolstered in subsequent computational analyses employing, e.g., finite element spring networks (Wilson and Bachofen, 1982; Makiyama et al., 2014; Albert et al., 2019) or systems of differential equations (Ma et al., 2023) to show that stress accumulates heterogeneously in the lung parenchyma, with the largest stresses found near areas with greater extents of injury. Other spring network simulations have shown that tethering (or stiffening) has both localized and longer length-scale effects on the distribution of lung stress and strain (Ma et al., 2013; Ma et al., 2015; Hall et al., 2023). Probabilistic methods, based on experimental data, have also been employed to understand the forces contributing to injury propagation, the mechanisms of injury heterogeneity, and the rich-get-richer mechanisms of VILI pathogenesis and offer a complementary perspective to deterministic mechanical models (Mattson et al., 2022; Mattson and Smith, 2023). That stochastic approach reaffirms the local to global range of injury interdependencies that emerge in a lung subjected to injurious ventilation. Collectively, this body of research shows how the presence of existing parenchymal injury alters the stress environment and drives the genesis of subsequent injury, supporting the conceptual framework of the current study. The spring network simulations provide a mechanism for injury propagation while the probabilistic models support this concept using *in vivo* data.

Our works builds upon these established theories by drawing an analogy to seismology, specifically the concept of aftershocks clustering around a main seismic event, to provide a novel perspective on lung injury propagation. The heterogeneity of forces within the lung may be viewed analogously to the heterogeneous redistribution of mechanical stress along geological fault lines, leading to asymmetric rupture propagation (Araki et al., 2006; Zaliapin and Ben-Zion, 2011). The dynamics of edema and pulmonary surfactant may also relate to the aftershock concept. In edematous areas of injury, high surface tension liquid might be ‘squeezed out’ into adjacent airspaces on expiration, spreading aftershocks of injury into adjacent regions (Perlman, 2020). Considering the sub-hour timescale of the experiments, inflammatory processes are unlikely to play a dominant role in the current study. Regardless of the exact mechanisms at play here, the analogy to seismology, particularly the concept of aftershocks clustering around a main seismic event, offers a vivid illustration of how lung injury might propagate. Just as aftershocks extend the impact of an earthquake, secondary injury sites can exacerbate and extend the damage initiated by a primary injury site. Recognizing this pattern enables us to conceptualize lung injury in a new light, offering insights into the mechanisms driving injury propagation in the lung.

Our study, while providing novel insights into the pathophysiology of acute and ventilator induced lung injury, has several limitations. First, the use of a simulated time series to model the progression of lung injury allows us to infer temporal dynamics that are otherwise unmeasurable. Our adoption of scale-free modeling and simulated time series analysis rests on simplifying assumptions that conceptualize the size and temporal development of lung injuries as independent variables. This perspective facilitates our exploration of injury distribution and progression patterns but may not fully capture the *in vivo* development of lung injury where it may be that larger injured regions form preferentially at later timepoints. Given that this information is currently unknown, we elected to maintain independence between injury size and temporal development. Nevertheless, the simulated time series results are consistent across the 100 different simulations in the ensemble and thus likely provide a reasonable approximation. Furthermore, in the simulated time series the regions of injury are generated instantaneously which does not capture the temporal growth of injury size, a process that may include adjacent injured regions merging with one another. In order to accommodate these factors, a spatially defined model framework would need to be employed and additional mechanistic or probabilistic factors would be necessary to drive injured region growth. Given the additional complexity and parameterization requirements posed by these additions we propose that the current approach provides a good balance between simplicity, feasibility, and accuracy. Reflecting on these assumptions, we recognize the need for caution in extrapolating our findings to clinical scenarios. Second, our reliance on 2D analysis for evaluating lung injury may oversimplify the inherently complex 3D structure of the lung and injured regions. This dimensional reduction could potentially obscure important spatial relationships and injury dynamics. The experimental challenges in 3D imaging at the whole-lung scale and necessary image resolution are formidable and will hopefully be addressed in years to come. Third, training algorithms to differentiate injured from healthy regions of the lung parenchyma is challenging. This is exacerbated by the limited staining options in glycol methacrylate sections which were chosen to avoid the dramatic shrinkage of lung tissue that occurs in paraffin processing (Schneider and Ochs, 2014).

In summary, automated segmentation of histological sections shows a power-law distribution of the size of regions of microatelectases and edema (injury) in the lung parenchyma of controls and mice subjected to an injurious pulmonary lavage and a half hour of VILI. The application of a network theory approach derived from seismology demonstrates that the number of correlations received by injured regions (the in-degree distribution) follows a power-law distribution. This distribution is indicative of a scale-free network. Simulated time series analysis suggests that the ‘aftershocks’ of these injured regions could follow similar patterns to earthquakes, where larger injured regions spawn more numerous secondary injury events in nearby regions of the parenchyma. Taken in the context of prior studies showing power-law distributions of perforations in the blood-gas barrier and pulmonary cell injury, the current findings suggest that the rich-get-richer mechanism of lung injury is present across a wide range of length scales and may govern the spatiotemporal heterogeneity that characterizes acute and ventilator-induced lung injury.

## Data Availability

Data is available from the corresponding author on reasonable request. Requests to access these datasets should be directed to bradford.smith@ucdenver.edu.
