# Supplementary material for: A scale-free model of acute and ventilator-induced lung injury: a network theory approach inspired by seismology
Source: Front Netw Physiol. 2024 May 1;4:1392701. doi: 10.3389/fnetp.2024.1392701 (PMC11097687; doi:10.3389/fnetp.2024.1392701)
Supplement: Supplementary file 1 [file DataSheet1.PDF]

A Scale-Free Model of Acute and Ventilator-Induced Lung Injury: A Network Theory Approach Inspired by Seismology

Drew C. Gottman, BS[1], Bradford J. Smith, PhD[2,3*]

[1]University of Colorado School of Medicine, University of Colorado Denver | Anschutz Medical Campus, Aurora, Colorado, USA

[2]Department of Bioengineering, University of Colorado Denver | Anschutz Medical Campus, Aurora, Colorado, USA.

[3]Section of Pulmonary and Sleep Medicine, Department of Pediatrics, School of Medicine, University of Colorado Denver | Anschutz Medical Campus, Aurora, Colorado, USA

Supplementary Material

1 Proof of Scale-Free Network with Simulated Time Series

1.1 Introduction

We start by first determining the distribution of the time intervals between all pairs of regions i and j. Assume that the distribution of time intervals has finite mean and variance, which is a reasonable assumption given the finite duration of the experiments. We also assume that in finding the product distribution of the corresponding random variables $N = N^* * T$, that N^* and T are statistically independent. We denote time intervals between successive i and j (i.e. $j = i + 1$) as $X_1, X_2, X_3, \dots, X_n$ with,

$$X = \{X_1, X_2, X_3, \dots, X_n\}, \tag{1}$$

We can get the value of a time interval between any i and j by summing successive time intervals together as follows:

$$T_{ij} = \sum_{a=i}^{j-1} X_a \,, \tag{2}$$

1.2 M-Dependence of Time Intervals

Next, we aim to compute the distribution of time intervals added together for a specific number of consecutive occurrences, and subsequently form a combined distribution from these individual distributions. For example, we'll determine the distribution for the sum of two consecutive time intervals, then three, four, and so on. By doing this, we can derive a composite distribution of the time intervals for all possible pairs of injury events i and j, allowing us to ultimately derive the distribution of $n_{ij} = n_{ij}^{*} * t_{ij}$. We will show that the distributions of M successively summed time intervals asymptotically approach the normal distribution with expected value $\mu = (M + 1) * \bar{X}$, where $\bar{X}$ is the expected value of the set $\bar{X}$.

We start by defining T^M as the set containing all $M + 1$ successive summations of X $(e.g. T^2 = \{X_1 + X_2 + X_3,\ X_2 + X_3 + X_4,\ X_3 + X_4 + X_5, \ldots, X_{n-2} + X_{n-1} + X_n\} = \{T_1^2,\ T_2^2, \ldots, T_{n-2}^2\}$). We note that each T_k^M and T_l^M will share terms in their summation if $|k - l| \leq M$; thus, T_k^M and T_l^M are dependent if $|k - l| \leq M$ and independent if $|k - l| > M$. Thus, the sequence of random variables in T^M are M-dependent.

1.3 Application of Basic Approximation Theorem

We use an adapted proof from *Time Series Analysis and Its Applications* to prove each T^M will have an approximately normal distribution (1). We will construct an approximation Y_{mn} of T^M and show that Y_{mn} asymptotically approaches the limiting distribution of T^M. In order to show this, we need to satisfy the three basic criteria of the basic approximation theorem for M-dependent sequences (1). These criteria are:

i. $y_{mn} \xrightarrow{d} y_m$ as $n \to \infty$ for each m
ii. $y_m \xrightarrow{d}$ y as $m \to \infty$
iii. $E\{|T_{n-M}^M - y_{mn}|^2\} \to 0$ as $m, n \to \infty$

We start by defining the autocovariance function of X, which is given by

$$\hat{\gamma}(h) = n^{-1} * \sum_{t=1}^{n-h} (X_{t+h} - \bar{X}) * (X_t - \bar{X}), \tag{3}$$

with $\hat{\gamma}(-h) = \hat{\gamma}(h)$ for $h = 0, 1, 2, \ldots, n - 1$. Note that because X is composed of independently and identically distributed random variables, that $\hat{\gamma}(h) = 0$ for all $h \neq 0$. If $h = 0$, then we simply get the variance of X.

We can also use Eq. 3 to compute the autocovariance of T^M. Note that because the autocovariance of T^M depends only on the difference between two values and because the expected value of T^M does not depend on where we are in the sequence, this implies that T^M is *weakly stationary*, which we will refer to as *stationary* henceforth. Similarly, X is stationary because it is independently and identically distributed.

We can then compute the variance of T^M as follows:

$$var(T^M) = E[(X_1 + X_2 + \cdots + X_{M+1})^2] - E[X_1 + X_2 + \cdots + X_{M+1}]^2$$
$$= E[X_1^2 + X_2^2 + \cdots + X_{M+1}^2 + 2X_1X_2 + 2X_1X_3 + \cdots + 2 * X_M X_{M+1}]$$
$$- (\mu_1 + \mu_2 + \cdots + \mu_{M+1})^2$$

For $i \neq j, E\left[X_i X_j\right] = \hat{\gamma}_{ij} + \mu_i \mu_j$. For $i = j, E\left[X_i X_j\right] = \hat{\gamma}_{ii} + \mu_i^2$. We can then rewrite the variance as:

$$var(T^m) = \sum_{i=1}^{M+1} \hat{\gamma}_{ii} + \sum_{i=1}^{M+1} \mu_i^2 + \sum\nolimits_{i \neq j} 2 * (\hat{\gamma}_{ij} + \mu_i \mu_j) \tag{4}$$
$$- \left(\sum_{i=1}^{M+1} \mu_i^2 + 2 * \sum_{i \neq j} \mu_i \mu_j \right)$$
$$= \sum_{i=1}^{M+1} \hat{\gamma}_{ii} + 2 * \sum_{i \neq j} \hat{\gamma}_{ij}$$
$$= (M+1) * var(X) = (M+1) * \sigma_X^2$$

where $h = |i - j|$. Note that Eq. 4 is true because both X and T^M are stationary. Stationarity also implies that the variance of T^M can be given a summation of X_i from an arbitrary starting value. A starting value of $i = 1$ is chosen for convenience.

Next, we standardize T^M to have zero mean and variance 1 by

$$\frac{T^M - (M+1) * \mu_x}{\sigma_x * \sqrt{M+1}} \quad ,$$

and then construct a set of Y_{mn} approximating the standardized T^M. For $m > 2M$, we may consider the approximation:

$$W_{mn} = \{(X_1 + X_2 + \cdots + X_{m-M-1}), (X_{m+1}, X_{m+2}, + \cdots \tag{5}$$
$$+ X_{2m-M-1}), (X_{2m+1} + X_{2m+2}$$
$$+ X_{3m-M-1}), \dots, (X_{(r-1)m+1} + X_{(r-1)m+2}$$
$$+ X_{rm-M-1})\}$$
$$Y_{mn} = \frac{W_{mn} - (m - M - 1) * \mu_x}{\sigma_x * \sqrt{m - M - 1}}$$
$$= \{z_1, z_2, z_3, \dots, z_r\}$$

where $r = \left[\frac{n}{m}\right]$, with $\left[\frac{n}{m}\right]$ denoting the greatest integer less than or equal to $\frac{n}{m}$. The approximation only contains part of T^M, but the random variables $z_1, z_2, z_3, \dots, z_r$ are independent because they are separated by more than M time points. Because of stationarity, they are identically distributed with mean 0 and variance 1 by similar calculation to Eq. 4. We are now ready to verify that the conditions of the basic approximation theorem hold.

i. Since each element of Y_{mn} is composed of sums of random variables that are independently and identically distributed with mean 0 and variance 1, we can apply the central limit theorem to find its limiting distribution. According to the central limit theorem:

$$Y_{mn} \overset{d}{\to} Y_m \sim S_{m-M-1} \approx N(0,1) \text{ as } n \to \infty \text{ for fixed } m,$$

with S_{m-M-1} the distribution of standardized sums with $m - M - 1$ terms. The approximation of S_{m-M-1} to $N(0,1)$ will improve as the number of terms in each sum becomes large.

ii. This brings us to the next part of the proof. According to the central limit theorem:

$$Y_m \sim S_{m-M-1} \overset{d}{\to} Y \sim N(0,1) \text{ as } m \to \infty ,$$

because the distribution of standardized sums asymptotically approximates the normal distribution as the number of terms in each sum increases.

iii. To verify the final condition, we need to show that $E\{|T_{n-M}^M - y_{mn}|^2\} \to 0$ as $m, n \to \infty$. We first note that:

$$\begin{aligned} E\{|T_{n-M}^M - Y_{mn}|^2\} &= var(|T_{n-M}^M - Y_{mn}|) + E\{|T_{n-M}^M - Y_{mn}|\}^2 \\ &= var(|T_{n-M}^M - Y_{mn}|) , \end{aligned}$$

since their expected values are both 0; thus, proving the third condition is equivalent to proving that the variance of $|T_{n-M}^M - Y_{mn}| \to 0$ as $m, n \to \infty$.

First denote the variance of the *standardized* Y_{mn} as $\sigma_{Y_{mn}}^2$ and the variance of the *standardized* T_{n-M}^M as $\sigma_{T_{n-m}^M}$. Now we have

$$\begin{aligned} var(|T_{n-M}^M - Y_{mn}|) &= \sigma_{Y_{mn}}^2 + \sigma_{T_{n-m}^M} - 2 * \hat{\gamma}_{TY} \\ &= \sigma_{Y_{mn}}^2 + \sigma_{T_{n-m}^M} - 2 * \sigma_{T_{n-m}^M} , \end{aligned}$$

with $\hat{\gamma}_{TY} = \sigma_{T_{n-m}^M}$ because the sums only overlap for the first $M + 1$ terms and the sums are composed of independent and identically distributed variables. We know that $\sigma_{Y_{mn}}^2 \to 1$ as $m, n \to \infty$ from parts one and two of the proof and that $\sigma_{T_{n-m}^M} \to 1$ as $m, n \to \infty$ because the variance only depends on M and because T^m is stationary, so the convergence of the

variance will not change with increasing sample size (controlled by n). Thus, the above expression reduces to 0 and condition three is satisfied. ■

This proof demonstrates that the standardized distribution of T^M can be approximated by $N(0,1)$, which implies that we can recover the original $T^M \dot{\sim} N[(M+1) * \mu_0,\ \sigma_M^2]$, where μ_0 is the mean of X and σ_M^2 is the variance of the samples in T^M.

1.4 Distribution of Link Weights

While the population variance of each T^M was equal to $(M+1) * \sigma_X^2$ in our construction, the sample variance is constrained by the fact that we have a single longest interval that limits the number of samples we can draw for each T^M. Specifically, the variance of T^{M-K} is equal to the variance of T^{K-2} for $K \geq 2$. This is because T^M is composed of a finite number of samples from an interval of total length $T^{n-1} = \{X_1 + X_2 + \cdots + X_n\}$. For example, T^{n-1} has sample variance of 0 since there is only one element in that set – the total length of the interval in which injuries occurred. T^0 and T^{n-2} have the same sample variance since T^0 is composed of individual elements of X, and T^{n-2} is composed of sums of $n-1$ consecutive elements of X. These two samples that are $n-1$ consecutive sums of X leave out X_n and X_1 from each of those sums, respectively. Thus, the sample variance of T^{n-2} is approximately equal to the sample variance of T^0. We can continue to apply this logic, noting that the sample variance of T^{n-3} is approximately equal to the sample variance of T^1, and so on. Thus, the sample variance of the entire set of all T^M is symmetric around $M = \frac{n-2}{2}$.

Now we aim to create a probability density function of T by summing the probability density functions of T^M for all values of M.

$$\begin{aligned} g_T(t) &= n * g_{T_0}(t) + (n-1) * \mathrm{g}_{\mathrm{T}_1}(t) + (n-2) * g_{T_2}(t) + \cdots \\ &\quad + g_{T_{n-1}}(\mathrm{t}) \\ &= n * N(\mu_0, \sigma_0^2) + (n-1) * N(2 * \mu_0, \sigma_1^2) + (n-2) \\ &\quad * N(3 * \mu_0, \sigma_2^2) + \cdots + N(n * \mu_0, \sigma_{n-1}^2), \end{aligned} \tag{6}$$

where $g_i(t)$ is the probability density function of T_i.

For simplicity, we modify Eq. 6 so that each function is weighted equally in the total sum. We will first examine this equally weighted sum before returning to the weighted sum. We denote the unweighted sum by:

$$\begin{aligned} g_T^*(t) &= N(\mu_0, \sigma_0^2) + N(2 * \mu_0, \sigma_1^2) + N(3 * \mu_0, \sigma_2^2) + \cdots + (n * \mu_0, \sigma_{n-1}^2) \\ &= \sum^{n-1} N(t \vert\ \mu_i, \sigma_i^2) \end{aligned} \tag{7}$$

$$= \sum_{i=0}^{n-1} N(\mu_i |\, t, \sigma_i^2).$$

Note that to get the last term in Eq. 7, we swapped the μ_i and t. This is possible because the value of the normal distribution depends on the difference between μ_i and t, and because the variance, σ_i^2, is symmetric around $i = \frac{n-2}{2}$ as noted previously. Thus, Eq. 7 gives us a sliding sum of a $N(\mu_i |\, t, \sigma_i^2)$ over the entire interval of normal distributions and will be approximately the same for all t because $N(\mu_i |\, t, \sigma_i^2)$ is symmetric around t. Thus $g_T^*(t) \propto U(\mu_0, n\mu_0)$, where $U(\mu_0, n\mu_0)$ is the uniform distribution over the interval bounded by μ_0 and $n\mu_0$.

This implies that $g_T(t) = -a * t + c$, where a and c are constants chosen to fit the above equation and t is a randomly chosen time interval from T. Notice that the normalization of the distribution to obtain total probability of 1 can be done by simply adjusting a and c.

Now to get the distribution of $n_{ij} = n_{ij}^* * t_{ij}$, we simply compute the product distribution $N = N^* * T$ where $h(n), f(n^*), g(t)$ define the probability distributions of distributions N, N^*, and T respectively. Recall that $f(n^*) = k * n^{*-b}$ because it is power − law distributed and $g(t) = -a * t + c$ from Eq. 7. This is done by first defining the cumulative density function of N:

$$H(N) = P(N \leq n) = P(N^* * T \leq n).$$

We can then calculate the probability density function by taking the derivative of $H(n)$ with respect to n:

$$h(n) = \frac{dH(n)}{dn}.$$

To find $H(n)$, we perform the double integral over the joint distribution of N^* and T:

$$H(n) = \iint D\, f(n^*) * g(t)\, dn^*\, dt,$$

where D is the domain in which $N^* * T \leq n$. Since $n^* * t \leq n$, we have $n^* \leq \frac{n}{t}$. This means that $D = \left\{(t,\ n^*) : 0 < t < \frac{c}{a}, 0 < n^* < \frac{n}{t}\right\}$ Now we can perform the integration

$$H(n) = \int_0^{\frac{c}{a}} \int_0^{\frac{n}{t}} (-a * t + c) * k * n^{*-b}\, dn^* dt$$

First, we integrate with respect to n^*:

$$\int_0^{\frac{n}{t}} (-a*t+c)*k*n^{*-b} dn^*$$
$$= (a*t-c)*k*\frac{\left(\frac{n}{t}\right)^{1-b}}{b-1}$$

Now, we obtain the integral with respect to t:

$$H(n) = k*\frac{n^{1-b}}{1-b}\int_0^{\frac{c}{a}} \left(\frac{1}{t}\right)^{1-b} (-a*t+c)\, dt$$

Notice that the integral with respect to t will simply be a constant that doesn't depend on n or the other random variables t and n^*. We simply group this integral with other constants that we denote by the collective C. Now, we differentiate $H(n)$ with respect to n to recover $h(n)$

$$\frac{dH(n)}{dn} = h(n) = C*n^{-b}. \tag{8}$$

Thus, we have shown that the probability density function of the product distribution $N = N^* * T$ also follows a power-law distribution. This means that the link weights between injured areas are power-law distributed across space and time.

1.5 Degree Distribution

Now, we must show that the degree distribution $P_{aggregate}(k) = k^{-a}$, where k is the degree of a given node in the network. We had previously shown in Eq. 8 that the distribution of the link weights between injured areas follows the form $h(n) = C*n^{-b}$. The cumulative distribution function (CDF) of $h(n)$ is given by:

$$H(n) = 1 - C\int_n^{\infty} t^{-b} dt$$
$$= 1 - C\frac{t^{1-b}}{1-b}\Bigg|_n^{\infty}$$
$$= 1 - C\left(0 - \frac{x^{1-b}}{1-b}\right)$$
$$= 1 + C*\frac{n^{1-b}}{1-b}\ ,$$

with the assumption that $b > 1$.

In order for node i to be connected to node j, node i must satisfy $\min(n_{ij})$. The probability that a given node i satisfies this is:

$$P_i = \int_{x_{\min}}^{\infty} h(x)\big(1 - H(x)\big)^{N-1} dx$$
$$= \int_{x_{\min}}^{\infty} C * x^{-b} \left(1 - 1 + C * \frac{x^{1-b}}{1-b}\right)^{N-1} dx$$
$$= C * \left[\left(\frac{x^{1-b}}{1-b}\right)^{N} * \frac{1}{N}\right]_{x_{\min}}^{\infty}$$
$$= \left(C * \frac{x_{\min}^{1-b}}{1-b}\right)^{N} * \frac{1}{N} \ , \qquad (9)$$

where N are the total number of nodes to select from and $x_{\min}$ is the smallest possible link weight.

From Eq. 9, we can see that the probability a given node i is connected to node j depends on the total nodes N to select from. For instance, imagine that we are looking at the second node in the time series. Then that node will be correlated to the first node with probability 1, and the expected number of connections is 1. Now imagine that we have two nodes in our pool to choose from. Since each node has an equal chance of being selected, the first node will have an expected number of connections of $3/2$, calculated by summing 1 and $1/2$, while the second node in the sequence will have an expected number of connections of $1/2$. Then, we can write the expected number of connections per node for the entire network as follows:

$$E[k] = \frac{H_N + (H_N - 1) + \left(H_N - 1 - \frac{1}{2}\right) + \cdots + (H_N - H_{N-1})}{N} \qquad (10)$$
$$= \frac{\mathrm{N} * \mathrm{H_N} - \mathrm{H_1} - \mathrm{H_2} - \cdots - \mathrm{H_{N-1}}}{N}$$
$$= \frac{\mathrm{N} * \mathrm{H}_N - \sum_{i=1}^{N-1} H_i}{N}$$
$$= \frac{\mathrm{N} * \mathrm{H}_N - \mathrm{N} * (\mathrm{H}_N - 1)}{N}$$
$$= 1,$$

where N is the total number of injured regions that can receive a correlation (equal to the total number of correlations in the network), k is the number of correlations per node, and H_N is the Nth harmonic number. Eq. 10 demonstrates that the average number of connections per node across the entire network is expected to equal 1. The intuition for this is clear since the total number of connections in the network is equal to N and there are N nodes that can receive a correlation.

Since we know that $E[k] = \int_1^N x * f(x)\, dx$, where $f(x)$ is the probability density function of the degree for each node, from Eq. 10, we can say that

$$1 = \int_1^N x * f(x)\, dx. \quad (11)$$

In the context of scale-free networks, which are known to exhibit an asymptotic power-law distribution of degrees, we extend Eq. 11 to consider an infinite network size an infinite size, where $N \rightarrow \infty$. This yields the following constraint:

$$1 = \int_1^\infty x * f(x)\, dx.$$

This integral implies that for the expected value to converge to a finite number, namely 1, the probability distribution function, $f(x)$, must decay more rapidly than $\frac{1}{x^2}$ for the expected value to converge to a finite value. Thus, the probability density function of the degree of the network is approximately power-law distributed and the network is scale-free.

2 Image Features Used for Pixel Classification

Supplementary Table 1: Image features used for pixel classification in Ilastik. Sigma (columns) defines the size of the Gaussian which is used to smooth the images prior to application of the filter (row). A larger sigma includes a greater area of the image, but averages out fine details.

	Sigma							
	0.7	2	5	10	25	50	75	100
Gaussian Smoothing		✓	✓	✓	✓	✓	✓	✓
Laplacian of Gaussian	✓	✓	✓	✓	✓	✓	✓	✓
Gaussian of Gradient Magnitude	✓	✓	✓	✓	✓	✓	✓	✓
Difference of Gaussians	✓	✓	✓	✓	✓	✓	✓	✓
Structure Tensor Eigenvalues	✓	✓	✓	✓	✓	✓	✓	✓
Hessian of Gaussian Eigenvalues	✓	✓	✓	✓	✓	✓	✓	✓

3 Supplementary References

1. Shumway RH, Stoffer DS. Time Series Analysis and Its Applications (Springer Texts in Statistics): Springer-Verlag; 2005.
